# Indeterminate Biliary Strictures: A Retrospective Study

**DOI:** 10.3390/jcm14113797

**Published:** 2025-05-28

**Authors:** Piotr Nehring, Magdalena Ciszewska, Adam Przybyłkowski

**Affiliations:** 1 Department of Gastroenterology and Internal Medicine, Medical University of Warsaw, Banacha 1a, 02-097 Warsaw, Poland; piotr.nehring@wum.edu.pl; 2 Faculty of Medicine, Medical University of Warsaw, Banacha 1a, 02-097 Warsaw, Poland

**Keywords:** biliary, cholangiopancreatography, endoscopy, indeterminate, stricture

## Abstract

**Background/Objectives:** Diagnosing biliary obstructions is challenging, especially when histopathology is inconclusive. Non-malignant biliary strictures often require additional tests and a personalized approach. This study investigates the prevalence, characteristics, and natural history of indeterminate biliary strictures. **Methods:** A retrospective analysis was conducted on 510 treatment-naive patients with hyperbilirubinemia due to biliary strictures or obstruction, who were all candidates for endoscopic retrograde cholangiopancreatography (ERCP). Patients with a known etiology before the procedure were excluded. Diagnosis was made via brush cytology or intraductal biopsy during ERCP, with follow-up for indeterminate cases. Statistical analysis was performed with Statistica software (version 13.3; TIBCO Software Inc. (2017), Palo Alto, CA, USA). **Results:** Out of 510 patients, 186 (36.5%) had non-malignant biliary strictures. Strictures were located in the liver hilum (29.6%), common bile duct (11.8%), and peripancreatic ducts (58.1%). Follow-up ERCP identified malignancy in 21.5% of cases initially deemed benign. Non-malignant causes were confirmed in 41.4% of initially benign strictures, while 37.1% remained indeterminate. After six months, 25.8% of cases remained unresolved. **Conclusions:** A quarter of benign biliary strictures remain indeterminate despite follow-up, and 20% are later identified as malignant. Improved diagnostic protocols are needed to better manage and expedite the diagnosis of indeterminate biliary strictures.

## 1. Introduction

The etiology of biliary obstruction can be divided into malignant (neoplastic) and non-malignant (benign). The true incidence of benign biliary strictures (BBS) is difficult to assess, as most of the studies focus on certain etiologies instead of the whole group. There are several causes of benign biliary obstruction, which can be grouped into iatrogenic, inflammatory, ischemic, infectious, autoimmune, and others [1]. The majority of BBS form after surgical procedures (laparoscopic cholecystectomy, biliary tract surgery, liver transplantation) or due to inflammation (i.e., chronic pancreatitis, acute pancreatitis) [2]. Other causes of benign biliary obstruction are cholelithiasis, primary sclerosing cholangitis (PSC), hepatic artery stenosis or thrombosis, radiotherapy, IgG4-related cholangiopathy, portal biliopathy, HIV cholangiopathy, tuberculosis, sarcoidosis, arteritis and lupus erythematosus, chemotherapy drugs, Oddi sphincter dysfunction or papillary stenosis, endoscopic retrograde cholangiopancreatography (ERCP)-related strictures, and liver transplantation [2]. The diagnosis of benign stenosis is based on the correlation of the patient’s medical history and imaging tests such as abdominal ultrasound (US), computed tomography (CT), magnetic resonance imaging (MRI), and endoscopic procedures, i.e., endosonography (EUS) and ERCP [1,2]. It is important to determine the proper nature of the stenosis to avoid the failure to recognize malignant stenosis in benign stenosis and surgery on benign lesions resembling malignant ones [3]. Before stepping to the endoscopic approach, available laboratory-based tests are routinely performed. However, the diagnostic performance of a common serum carbohydrate antigen 19.9 (CA 19.9) concentration is limited. In about 10% of the population, CA 19.9 is not produced due to the Lewis antigen negativity [4]. Moreover, CA 19.9 concentration may be elevated by the bile duct obstruction and cholangitis themselves, making it impossible to distinguish between neoplasms and BBS [5].

To exclude a neoplastic cause of biliary obstruction, histopathological examinations are used—brush cytology and intraductal biopsy. Navaneethan et al., in their meta-analysis, showed that both brushings and biopsy are comparable, and both have limited sensitivity for the diagnosis of neoplastic bile duct strictures [6]. In the aforementioned meta-analysis, brushings had 45% sensitivity and 99% specificity, whereas intraductal biopsies had 48.1% and 99.2%, respectively [6]. Advanced endoscopic techniques may be helpful in the diagnostic approach of indeterminate biliary strictures. Peroral cholangioscopy-guided, SpyGlass^TM^, sampling enables targeted biopsies and brushings, increasing diagnostic success [7]. EUS with needle aspiration is best at diagnosing pancreatic tumors, but not cholangiocarcinoma [8]. Moreover, EUS-guided needle aspiration may increase the risk of dissemination to the peritoneum in the case of hilar cholangiocarcinoma; therefore, its use in this indication is limited [9].

The premise for this study was a lack of data defining the prevalence of indeterminate biliary strictures, as the available literature is focused on certain etiologies of BBS. However, it may be estimated that 10–20% of cases of patients requiring therapeutic ERCP have indeterminate strictures [10,11,12]. There are no studies describing the natural history of indeterminate biliary strictures.

This study aimed to assess the prevalence, characteristics, and natural history of indeterminate biliary strictures.

## 2. Patients and Methods

### 2.1. Patients

The electronic records database of the Endoscopy Unit at the Department of Gastroenterology and Internal Medicine, a main endoscopy unit at the Medical University of Warsaw, Poland, was searched retrospectively for patients who had required primary biliary stenting due to strictures during ERCP between 2020 and 2022. Only patients who had primary ERCP were included. Patients after liver transplantation, with a visualized tumor mass, lithiasis, acute pancreatitis, primary sclerotizing cholangitis (PSC), and any known etiology before the ERCP procedure etiology visualized in imaging examinations (US, CT, MRI, or EUS), were excluded from this study. All patients with biliary stenting due to lithiasis without biliary stricture and with diagnosed bile duct or pancreatic malignancy before ERCP were not included.

The diagnosis of potential BBS was based on the first results of the first cytology or histopathology obtained from bile ducts that were negative for malignancy and the results of CT, MRI, or EUS. A negative result for malignancy was based upon the intraductal biopsy of lack of atypical or dysplastic cells. In brush cytology, as a result, excluding neoplasm was considered the result of benign/negative for malignancy, Category II, according to Papanicolaou Society of Cytopathology guidelines [13]. Patients with Category II, according to the Papanicolaou Society of Cytopathology, were further followed up in 2- to 3-month intervals as long as there was a need for an endoscopic bile duct strictures treatment (i.e., cholangiography, brush biopsies, imaging examinations, and serological markers), depending on clinical needs.

### 2.2. Statistical Analysis

All parametric variables were assessed for a normal distribution and variance. Student’s *t*-test and Mann–Whitney *U* test were used for parametric and nonparametric variables, respectively. For qualitative variables, the χ^2^ test was used, with Fisher’s correction for small samples. Missing data were removed in pairs. Statistical significance was set for α < 0.05. Statistical analysis was performed with Statistica software (version 13.3; TIBCO Software Inc. (2017), Palo Alto, CA, USA).

## 3. Results

This study enrolled 510 naive patients with biliary obstruction treated primarily with ERCP. Of these, 324 patients (63.5%) diagnosed with bile duct obstruction were established during the primary ERCP; therefore, this group was not further analyzed. In the other 186 (36.5%) patients, the primary cytology/histopathology report from the ERCP was benign/negative for malignancy (Category II according to the Papanicolaou Society of Cytopathology). In a group with malignant-negative biliary strictures, 55 (29.6%) strictures were localized in the liver hilum (bifurcation of right and left liver ducts, and in the common liver duct), 22 (11.8%) in the common bile duct, 108 (58.1%) in the intrapancreatic/peripapillary part of the bile ducts (Table 1).

**Figure 1:**
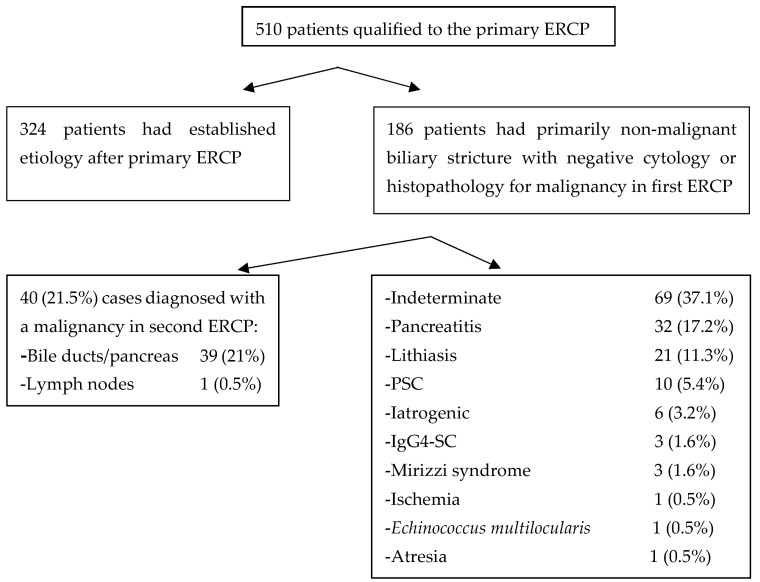
Chart of bile duct stricture etiologies in the study.

In the group of primarily BBS, subsequent ERCP malignant etiology has been proved in 40 patients; that is, 21.5%. Known, non-malignant etiologies (i.e., pancreatitis and its complications, PSC, ischemia, IgG4-SC, Mirizzi syndrome, and atresia) stand for 77 of cases; that is, 41.4% of cases. Sixty-nine of biliary strictures in subsequent ERCP remained indeterminate (of unknown origin); that is, 37.1% of the primarily BBS cases (Figure 1).

Comparing the localizations of the indeterminate biliary strictures, there were no differences in sex, age, body mass, weight, and height between patients. Cellular atypia in histological or cytological examination, and as a consequence, a malignancy in subsequent biopsies during ERCP, were more frequent in patients with biliary strictures localized in the liver hilum. Among clinical findings, concomitant cholelithiasis was less frequent in strictures localized in the CBD, and concomitant cholangitis was less frequent in peripancreatic localization. In the group of patients with non-malignant biliary stricture localized in the liver hilum, the most frequent etiology was malignancy in subsequent biopsies (30%) and truly indeterminate etiology (30%). In the group of patients with strictures localized in the CBD and in peripancreatic localization, the most frequent etiology was indeterminate, respectively, in 41% and 40% of cases. The malignant etiology primarily recognized as benign biliary stricture was set in 30% of patients with strictures in the liver hilum, 23% in the CBD, and 16% in peripancreatic localization.

Lithiasis was the statistically more frequent reason for benign biliary stricture in patients with bile duct narrowing localized in the peripancreatic part of the biliary tree compared to the CBD and liver hilum (12% vs. 4.5% vs. 0%, respectively). PSC was a more frequent etiology of bile duct narrowing in patients with biliary strictures localized in the liver hilum, comparing CBD and peripancreatic localization (14% vs. 4.5% vs. 0%, respectively) (Table 2).

Among the 69 patients initially presenting with indeterminate biliary strictures, 17 patients received a definitive diagnosis during the 6-month follow-up period. Specifically, eight patients were identified as having lithiasis as the primary cause, which was effectively resolved through stenting without recurrence. Five patients were diagnosed with cholangiocarcinoma (malignancy), two with chronic pancreatitis, one with a benign neoplasm (serous cystic adenoma of the biliary duct), and one with IgG-SC. Four patients passed away from unrelated causes before undergoing subsequent ERCP. The remaining 48 patients (25.8%) were unable to be definitively diagnosed within the 6-month follow-up period and continued to present with indeterminate biliary strictures.

In all five patients who were finally diagnosed with cholangiocarcinoma within six months since the first ERCP, diagnosis was set in subsequent ERCP with forceps biopsy (third or fourth procedure).

Out of the final 48 indeterminate cases in the next 12-month follow-up, 21 cases were lost from further follow-up, 12 cases were cured (did not require further stenting and there was no stricture recurrence), in 8 patients a tumor mass was visualized in imaging, although no malignancy was confirmed (highest cytology grade was V from FNA), and 7 patients died from other reasons.

**Figure 2:**
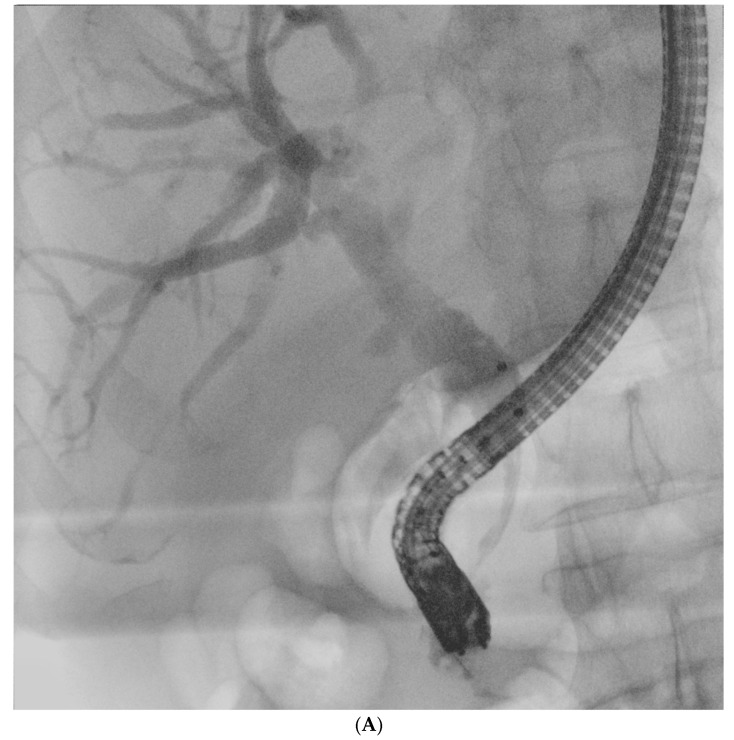

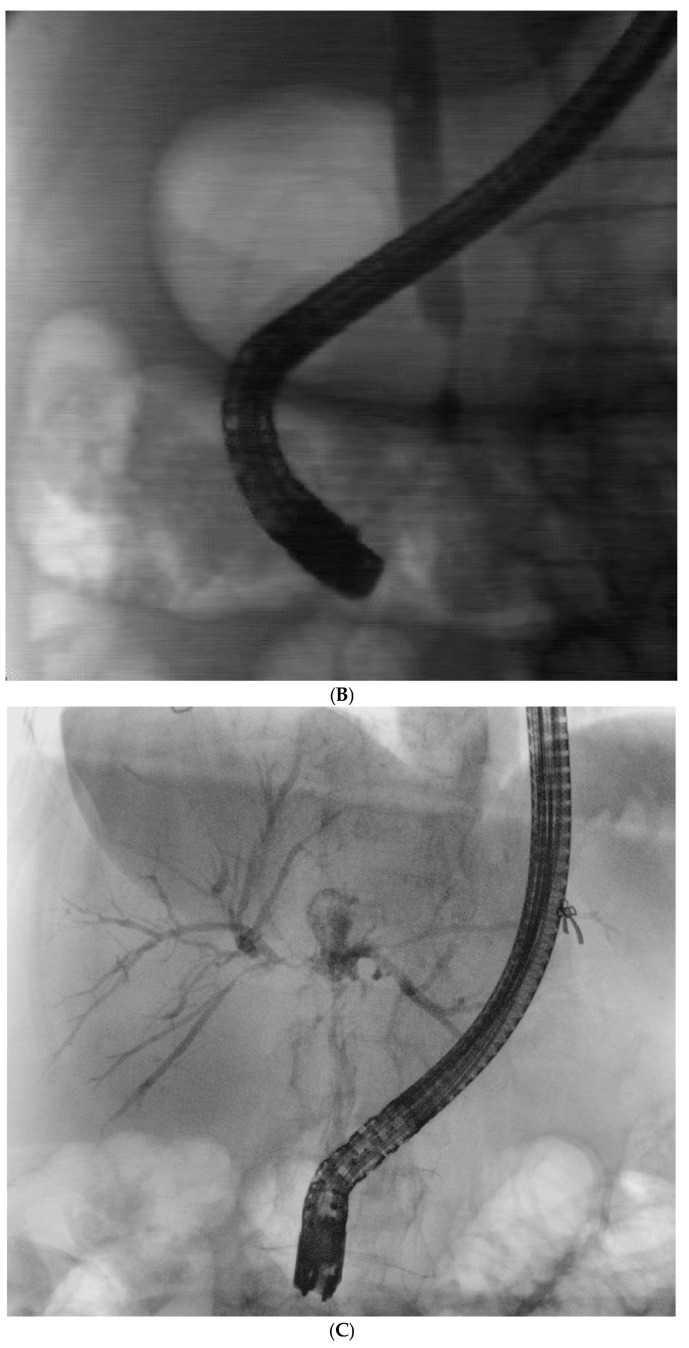

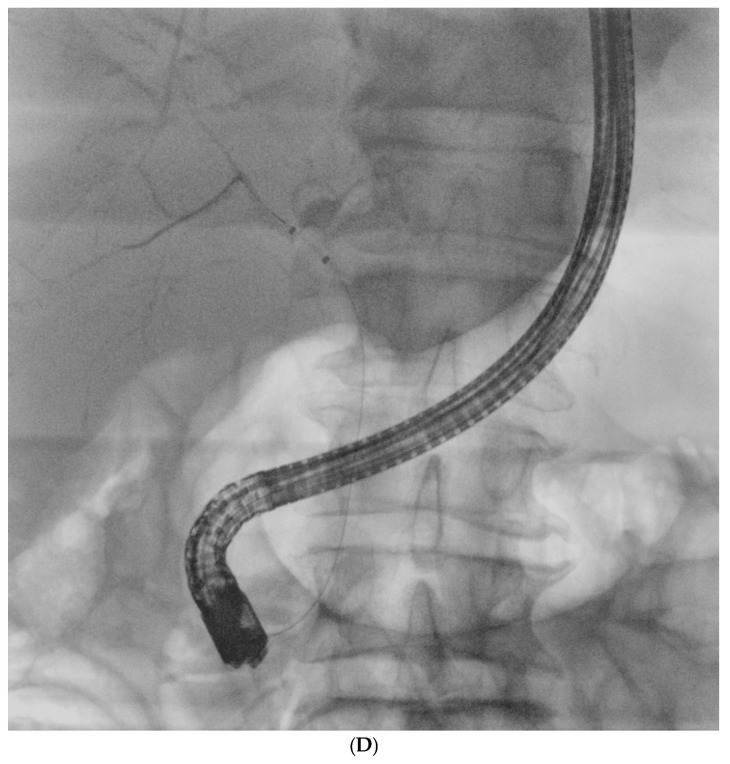
(**A**) The common bile duct stricture in transpancreatic segment (tumor of the pancreas). (**B**) Indeterminate stricture in the distal common bile duct. (**C**) Cholangiocarcinoma of liver hilum extending to the right and left hepatic ducts (Bismuth-Corlette IV). (**D**) Multiple strictures of bile ducts, a typical image in primary sclerosing cholangitis.

Figure 2A–D Cholangiograms of different etiology of bile duct strictures.

## 4. Discussion

The presented data substantiate that the etiology of primarily non-malignant biliary strictures identified during subsequent ERCP with brush cytology/histopathological biopsy may remain unknown in up to 25% of cases.

Some data on this topic can be retrieved from various studies on patients treated with ERCP. In a study by Draganov et al., evaluating the diagnostic accuracy of sampling methods during an ERCP long follow-up of 21.78 (SD ± 6.78) months, nine patients with indeterminate strictures were documented [7]. The final diagnosis was idiopathic stricture in three patients, inflammatory stricture in three individuals, and one case each of choledochal cyst, postoperative stricture, and granulomatous liver disease [7].

In our study, 29.6% of indeterminate strictures were localized in the liver hilum, 11.8% in the common bile duct, and 58.1% in the intrapancreatic/peripapillary part of the bile ducts, which is generally in line with other studies [2,3].

In the present study, malignant etiology based on subsequent ERCP of the primarily BBS in 21.5% of cases was proved, which is much below the expected sensitivity and specificity of brush cytology and forceps biopsy. In a meta-analysis of studies on the diagnostic accuracy of malignant biliary strictures, the sensitivity and specificity of brush cytology were 45% and 99%, respectively, and for forceps biopsy, they were 48.1% and 99.2%, respectively [6]. The combination of both brush biopsy and forceps biopsy improved sensitivity and specificity up to 59.4% and 100%, respectively [6]. In Nur et al.’s study, 26% of patients requiring brushing during primary ERCP had a negative result for malignancy, and 16% of patients with confirmed malignancy showed false negative results of brushing [10]. Interestingly, in the Alali et al. study, among patients with indeterminate biliary strictures with atypical cytology from brushing in primary ERCP, 52.3% of patients were diagnosed with malignancies in the subsequent biopsies [11]. This observed low sensitivity and specificity of brush cytology, and forceps biopsy in our study is probably a consequence of the exclusion of malignant cases diagnosed during primary ERCP from the analysis (selection bias).

In the present study, considering longer follow-up, frequent malignancy was still diagnosed. Therefore, patients with indeterminate biliary stricture require careful planning of the diagnostic approach and frequent monitoring with the collection of multiple biopsies.

Clinicians frequently encounter challenges in elucidating the etiology of non-malignant biliary strictures, and some cases remain indeterminate. Hence, it is imperative not only to conduct repeated brush cytology or histological biopsies during ERCP but also to perform imaging examinations and apply modern endoscopy techniques, such as cholangioscopy, in search of the etiology of stricture.

According to the six-tiered Papanicolaou classification system, a cytology result categorized as Category II indicates a specimen that is “negative for malignancy”. This designation represents the most favorable cytological outcome, suggestive—although not definitively confirmatory—of a benign etiology for the biliary stricture. Qualitatively, this category encompasses cases deemed “unsuspicious” for neoplastic diseases. In the present study, this type of patient follows the routine diagnostic approach of subsequent specimens taken during ERCP, imaging examinations (MRI, CT), or cholangioscopy, when necessary.

In contrast, Category I denotes a non-diagnostic or unsatisfactory specimen, whereas Categories III through VI range from atypical cells (III), suspicious for malignancy (IV), and positive for malignancy (V), to unequivocally malignant (VI) [12].

At our institution, all patients with Category III cytology findings were prioritized for expedited further diagnostic evaluation. This process was typically initiated within two weeks, considering that complete diagnostic data (including cytological and histopathological analyses) required approximately 10 days for processing.

Due to variability in the stage at which patients entered the diagnostic pathway, a standardized protocol was not uniformly applied. Nevertheless, in the majority of cases, the diagnostic approach was escalated using one or more of the following procedures: ERCP with brush cytology and/or intraductal biopsy, EUS with FNA, and peroral cholangioscopy with targeted forceps biopsy.

In the current European Society of Gastroenterology and Endoscopy (ESGE) guidelines on ERCP, there is a precise recommendation on how to treat BBS of known etiology, such as BBS after liver transplantation, post-cholecystectomy, post-sphincterotomy, or in chronic pancreatitis [14]. However, the guidelines lack diagnostic and follow-up for indeterminate strictures.

The most up-to-date algorithm was proposed by Yadlapati et al. [12]. In indeterminate biliary strictures, that is, after prior ERCP with tissue sampling, after in-detail history taking, laboratory testing, and prior imaging results revision, the further approach is based on biliary strictures localization. In cases of indeterminate biliary stricture localized in distal (below 2 cm from the biliary confluence) EUS +/− ERCP is performed based on the visibility of tumor mass. In cases of proximal strictures or localized in liver hilum, ERCP with brush biopsies and cholangioscopy are performed. In both situations, in the case of non-diagnostic results of subsequent investigations, Yadlapati et al. proposed using intraductal ultrasound (IDUS) or probe-based confocal laser micro-endoscopy (pCLE) [12].

A study by Varadarajulu et al. showed the high effectiveness of rapid onsite evaluation of touch imprint cytology (ROSE-TIC) for diagnosing malignancy in patients with primarily indeterminate biliary strictures [15]. The overall sensitivity of ROSE-TIC was 100%, specificity 88.9%, positive predictive value 86.7%, negative predictive value 100%, and diagnostic accuracy 93.5%.

Peroral cholangioscopy is advisable at the early stages of investigating indeterminate biliary structures due to secondary changes in bile duct tissue after stenting, which may compromise cytological or histological assessments. This issue was raised in Sethi et al.’s study, which shows that the reactive atypia resulting from the presence of a stent can give rise to equivocal cytological reports [16]. However, there are no direct studies on the topic of indeterminate biliary strictures.

Further monitoring in patients with biliary strictures of unknown etiology is particularly justified when the strictures persist and require stenting. In such cases, the intervals for stent replacement typically determine the timing of subsequent procedures, during which tissue samples are collected for histopathological examination. Importantly, this interval is usually shortened in patients suspected of a neoplastic or autoimmunological etiology, as establishing a definitive diagnosis prompts the initiation of appropriate causal treatment. Therefore, it is advisable not to perform ERCP procedures less frequently than every 2–3 months, ideally in conjunction with imaging studies.

An alternative diagnostic approach for biliary strictures involves the detection of neoplastic markers in bile obtained through intraductal aspiration during ERCP, a bile intraductal aspiration (BIDA). Both CA 19.9 and carcinoembryonic antigen (CEA) exhibit reasonable sensitivity for cholangiocarcinoma, with up to 74% for CA 19.9 and 84% for CEA [17]. There is an emerging role for new potential markers of carcinogenesis, such as neutrophil gelatinase-associated lipocalin (NGAL) and carcinoembryonic cell adhesion molecule 6 (CEAM6), vascular endothelial growth factor (VEGF), heat shock proteins (HSP) 27, and 70 insulin-like growth factor (IGF), and specific oxidized phospholipids (i.e., ON-phosphatidylcholine and S-phosphatidylcholine) assessed in BIDA aspirate [15,17,18,19].

Another alternative approach for BBS diagnosis is volatile organic compounds (VOC) analysis. In Navaneethan et al.’s study, it was demonstrated that the VOC signature in bile in the headspaces may be useful in differentiating pancreatic cancer from BBS, as well as cholangiocarcinoma in primary sclerosing cholangitis [20,21]. Navanethan et al. proposed the following algorithm for the diagnostics of indeterminate biliary strictures: ERCP with BIDA followed by implementation of proteomics or lipidomic and VOC examination [22]. Although serum CA 19.9 and CEA are not definitive for diagnosis of the etiology of biliary strictures, considering the diagnostic challenges of indeterminate biliary strictures, it is reasonable to monitor CA 19.9 and CEA values in addition to imaging and histopathology.

In summary, after follow-up and detailed examination, one-quarter of benign biliary strictures remain indeterminate, while one-fifth of those initially considered benign are found to be malignant within six months. There is a need to develop effective protocols and guidelines to enhance and expedite the diagnosis of primary benign bile duct strictures.

## Figures and Tables

**Table 1:** Characteristics of the studied population of patients with malignant-negative biliary obstruction (n = 186), obstruction localizations, and histopathology reports.

Feature	Result
Age, y, mean ± SD	65.1 ± 14.8
Sex n (%):	
- Males	106 (57)
- Females	80 (43)
Height, cm, mean ± SD	169.8 ± 10.1
Weight, kg, mean ± SD	70.7 ± 13.7
Stricture’s localization, n (%):	
- liver hilum	55 (29.6)
- common bile duct	22 (11.8)
- intrapancreatic	108 (58.1)
Cytology/histopathology, n (%):	
- Fibrosis	47 (22.7)
- Atypia	40 (19)
- Dysplasia	25 (12)
- Metaplasia	6 (3)
- Inflammation:	86 (41.5)
○ Neutrocytes	28 (13.5)
○ Plasmocytes	9 (4.3)
○ Lymphocytes	9 (4.3)
○ Eosinophils	1 (0.5)
- Malignancy (in subsequent ERCP)	37 (18)
Important clinical data and ERCP findings, n (%):	
- Concomitant cholelithiasis	130 (63)
- Concomitant cholangitis	95 (46)
- Cholecystectomy	65 (31)
Diagnosed biliary stricture etiology, n (%):	
- Indeterminate	69 (37.1)
- Bile ducts/pancreatic malignancy *	39 (21)
- Pancreatitis (chronic, or complications of acute)	32 (17.2)
- Lithiasis	21 (11.3)
- PSC	10 (5.4)
- Iatrogenic	6 (3.2)
- IgG4-SC	3 (1.5)
- Mirizzi syndrome	3 (1.5)
- Lymph node mass effect	1 (0.5)
- Ischemia	1 (0.5)
- *Echinococcus multilocularis*	1 (0.5)
- Atresia	1 (0.5)

SD—standard deviation; ERCP—endoscopic retrograde cholangiopancreatography; PSC—primary sclerotizing cholangitis; IgG4-SC—IgG4-related sclerotizing cholangitis; * results considered as being of malignant etiology.

**Table 2:** Comparison of the indeterminate stricture characteristics between their localizations in the biliary ducts.

	Liver Hilum N = 55	CBD N = 22	Intrapancreatic N = 108	*p* Value
Age, y, mean ± SD	61 ± 17	64.8 ± 15.8	67 ± 13	<0.17
Sex: Males/Females	32/23	14/8	59/49	<0.72
Height, cm, mean ± SD	171 ± 10	169 ± 11	169 ± 14	<0.43
Weight, kg, mean ± SD	73 ± 13	69.2 ± 12.5	69.5 ± 10	<0.45
Cytology/histopathology:	n (%)	n (%)	n (%)	
- Fibrosis	17 (30)	4 (18)	25 (23)	<0.49
- Atypia	23 (40)	2 (9)	15 (14)	<0.0001
- Dysplasia	9 (16)	2 (9)	14 (13)	<0.72
- Metaplasia	2 (3.5)	3 (13)	3 (2.8)	<0.90
- Inflammation:	24 (42)	5 (23)	55 (51)	<0.05
○ Neutrocytes	12 (21)	3 (13)	13 (12)	<0.30
○ Plasmocytes	4 (7)	0	3 (2.8)	<0.29
○ Lymphocytes	4 (7)	0	5 (4.6)	<0.42
○ Eosinophils	1 (1.8)	0	0	<0.32
Malignancy (diagnosed in second ERCP)	19 (33)	3 (13)	15 (14)	<0.01
Important clinical data and ERCP findings:				
- Concomitant cholelithiasis	37 (66)	8 (36)	69 (64)	<0.02
- Concomitant cholangitis	40 (70)	10 (45.5)	37 (34.5)	<0.001
- Cholecystectomy	19 (33)	8 (36)	35 (32)	<0.94
Diagnosed biliary stricture etiology:				
- Indeterminate	17 (30)	9 (41)	43 (40)	<0.41
- Lithiasis	0	1 (4.5)	20 (12)	<0.001
- Bile ducts/pancreatic malignancy *	17 (30)	5 (23)	17 (16)	<0.10
- Pancreatitis (chronic or complications of acute)	4 (7)	4 (18)	23 (21)	<0.06
- PSC	8 (14)	1 (4.5)	1 (1)	<0.002
- IgG4-SC	2 (4)	1 (4.5)	0	<0.12
- Lymph node mass effect *	1 (2)	0	0	<0.32
- Ischemia	1 (2)	0	0	<0.32
- Mirizzi syndrome	2 (4)	0	1	<0.37
- *Echinococcus multilocularis*	1 (2)	0	0	<0.32
- Iatrogenic	3 (6)	1 (4.5)	2 (2)	<0.70

SD—standard deviation; CBD—common bile duct; ERCP—endoscopic retrograde cholangiopancreatography; PSC—primary sclerotizing cholangitis SC—IgG4-related sclerotizing cholangitis; * results considered as being of malignant etiology.

## Data Availability

The data presented in this study are available on request from the corresponding author.
